# Microbiota dysbiosis in inflammatory bowel diseases: *in silico* investigation of the oxygen hypothesis

**DOI:** 10.1186/s12918-017-0522-1

**Published:** 2017-12-28

**Authors:** Michael A. Henson, Poonam Phalak

**Affiliations:** Department of Chemical Engineering and the Institute for Applied Life Sciences, University of Massachusetts, 140 Thatcher Way, Amherst, 01003 MA USA

**Keywords:** Inflammatory bowel disease, Gut microbiota, Biofilms, Metabolic modeling

## Abstract

**Background:**

Inflammatory bowel diseases (IBD), which include ulcerative colitis and Crohn’s disease, cause chronic inflammation of the digestive tract in approximately 1.6 million Americans. A signature of IBD is dysbiosis of the gut microbiota marked by a significant reduction of obligate anaerobes and a sharp increase in facultative anaerobes. Numerous experimental studies have shown that IBD is strongly correlated with a decrease of *Faecalibacterium prausnitzii* and an increase of *Escherichia coli*. One hypothesis is that chronic inflammation induces increased oxygen levels in the gut, which in turn causes an imbalance between obligate and facultative anaerobes.

**Results:**

To computationally investigate the oxygen hypothesis, we developed a multispecies biofilm model based on genome-scale metabolic reconstructions of *F. prausnitzii*, *E. coli* and the common gut anaerobe *Bacteroides thetaiotaomicron*. Application of low bulk oxygen concentrations at the biofilm boundary reproduced experimentally observed behavior characterized by a sharp decrease of *F. prausnitzii* and a large increase of *E. coli*, demonstrating that dysbiosis consistent with IBD disease progression could be qualitatively predicted solely based on metabolic differences between the species. A diet with balanced carbohydrate and protein content was predicted to represent a metabolic “sweet spot” that increased the oxygen range over which *F. prausnitzii* could remain competitive and IBD could be sublimated. Host-microbiota feedback incorporated via a simple linear feedback between the average *F. prausnitzii* concentration and the bulk oxygen concentration did not substantially change the range of oxygen concentrations where dysbiosis was predicted, but the transition from normal species abundances to severe dysbiosis was much more dramatic and occurred over a much longer timescale. Similar predictions were obtained with sustained antibiotic treatment replacing a sustained oxygen perturbation, demonstrating how IBD might progress over several years with few noticeable effects and then suddenly produce severe disease symptoms.

**Conclusions:**

The multispecies biofilm metabolic model predicted that oxygen concentrations of ∼1 micromolar within the gut could cause microbiota dysbiosis consistent with those observed experimentally for inflammatory bowel diseases. Our model predictions could be tested directly through the development of an appropriate in vitro system of the three species community and testing of microbiota-host interactions in gnotobiotic mice.

**Electronic supplementary material:**

The online version of this article (doi:10.1186/s12918-017-0522-1) contains supplementary material, which is available to authorized users.

## Background

Inflammatory bowel diseases (IBDs) include ulcerative colitis [1], which is restricted to the large intestine and rectum, and Crohn’s disease [2], which can affect the entire digestive tract from the mouth to the anus. While both diseases are characterized by inflammation of the epithelial lining, Crohn’s disease can affect all layers of the intestinal wall. Current estimates are that 1.6 million Americans suffer from IBD and 70,000 new cases are diagnosed each year [3]. Common treatments including anti-inflammatory drugs, immune system suppressors and antibiotics often have unpredictable impacts on disease progression, especially in the more difficult-to-treat Crohn’s disease [4]. Therefore surgery is a common treatment option, with 70–90% of patients suffering from Crohn’s disease ultimately requiring surgery [5]. The direct and indirect costs of IBD treatment in the U.S. were estimated as $14.6–$31.6 billion in 2014 [6].

The underlying cause of IBD pathogenesis is not well understood, with genetics, diet and environmental factors all believed to play important roles [7]. The human gut harbors a highly complex microbial community that allows the digestion of dietary fibers [8] and has a profound influence on immune system health [9]. The gut microbiome consists of approximately 10^14^ bacterial cells representing 1000 species with 30 times the genomic content of the human host [10, 11]. The two dominant phyla in healthy humans are Bacteroidetes and Firmicutes, which contain obligate anaerobes that comprise about 90% of the bacterial community [12]. These two phyla are largely responsible for converting dietary fiber into short-chain fatty acids (SCFAs) including acetate, propionate and butyrate, that can be absorbed by the host intestine [13]. In addition to being the preferred energy source of colonic enterocytes [14], butyrate is thought to have anti-inflammatory properties [15]. Other phyla are less prevalent but also play critical roles in microbiome function, including facultative anaerobes from the Proteobacteria phylum which are thought to provide colonization resistance against pathogenic bacteria [16].

The gut microbiome is usually robust to dietary patterns and environmental perturbations that would otherwise alter species compositions and SCFA levels. IBD is characterized by long-term changes in the gut microbiota that are correlated with intestinal inflammation [17, 18] through a poorly understood process known as dysbiosis [11]. A common signature of IBD is a severe reduction in butyrate producing obligate anaerobes from the Firmicutes phylum, the most abundant of which is *Faecalibacterium prausnitzii* [10, 15]. Another common feature is a large increase in facultative anaerobes from the Proteobacteria phylum, most notably *Escherichia coli*. By contrast, no clear trend has been established for some other important obligate anaerobes such as *Bacteroides thetaiotaomicron* from the Bacteroidetes phylum.

Because IBD dysbiosis is characterized by an imbalance between obligate and facultative anaerobes, oxygen and reactive oxygen species have been hypothesized to play a key role in the pathogenesis [19]. The “oxygen hypothesis” posits that chronic inflammation of intestinal walls results in increased release of hemoglobin carrying oxygen and reactive oxygen species into the intestinal lumen [20], which in turn creates a microenvironment that favors facultative anaerobes [21, 22]. The resulting decrease in obligate anaerobes such as *F. prausnitzii* that release anti-inflammatory compounds causes increased inflammation [23], establishing a positive feedback loop that accelerates the disease process [24]. Because obligate anaerobes typically are not viable at dissolved oxygen concentrations greater than 5 *μ*M [25], the oxygen levels at which dysbiosis occurs likely needs to be at sub-micromolar levels to ensure a functional population of obligate anaerobes. A recent study with wild-type mice demonstrated a mechanistic link between butyrate levels and oxygen consumption by colonocytes via the beta-oxidation pathway [26]. As buytrate was depleted through antibiotic-induced death of butyrate producing bacteria, oxygen consumption by colonocytes decreased and oxygen levels increased accordingly. However, direct experimental testing of the oxygen hypothesis remains difficult due to challenges in establishing suitable animal models [10], limited technology for accurate measurement of in vivo oxygen concentrations [19] and the inability of measure species level changes with deep sequencing methods [10].

As a complement to ongoing in vitro and in vivo investigations, in this study we developed an *in silico* model of a minimal bacterial community for studying IBD consisting of *F. prausnitzii*, *E. coli* and *B thetaiotaomicron*. The community was modeled as a multispecies biofilm attached to the intestinal mucosa [27–29] with nutrient competition, byproduct cross feeding and diffusion limited growth. The model was tuned to qualitatively reproduce phyla abundances and SCFA levels measured in healthy gut microbiomes. Then oxygen was applied at the biofilm boundary to determine if the community would dynamically evolve to the IBD phenotype characterized by decreased *F. prausnitzii* and increased *E. coli* abundances. We further explored the effect of diet on oxygen sensitivity and incorporated a simple linear feedback to investigate how host-microbiome interactions might amplify oxygen-mediated dysbiosis. Our “bottoms-up” modeling approach based on curated genome-scale metabolic reconstructions of the participating species complements previous “top-down” approaches in which metagenomic data and genome-scale metabolic modeling were integrated to delineate both gene-level and network-level topological differences between healthy and IBD patients [30, 31].

## Methods

### Biofilm model formulation

The bacterial community model was developed for a multispecies biofilm growing in a simulated gut environment under the common assumption that intracellular metabolism responded instantaneously to changes in the extracellular environment [32]. The biofilm model was built upon curated genome-scale metabolic reconstructions of *B. thetaiotaomicron* [33], *F. prausnitzii* [34] and *E. coli* [35]. The simulated media was developed by combining all nutrients required for growth of the three species with amino acids and simple carbohydrates expected in the gut. To maintain reasonable computational complexity, the media was limited to four monosaccharides (arabinose, fructose, galactose, glucose) and ten amino acids (cysteine, isoleucine, leucine, lysine, methionine, proline, serine, threonine, tryptophan, valine). These four sugars were included because they are major bacterial degradation products of more complex carbohydrates [36, 37] and they can be consumed by all three species. The amino acids were chosen because they were essential for *F. prausnitzii* growth (methionine, serine, tryptophan), commonly catabolized by gut bacteria (lysine, theoronine, valine) [38], and/or essential for growth of the gut pathogen *Clostridium difficile* (cysteine, isoleucine, leucine, proline) [39], which will be included in our future modeling studies. Cellular growth was assumed to be potentially limited only by the carbohydates, amino acids and oxygen. More detailed description of the simulated media and the uptake bounds imposed in the genome-scale reconstructions are presented in the next section and in Additional file 1.

**Fig. 1:**
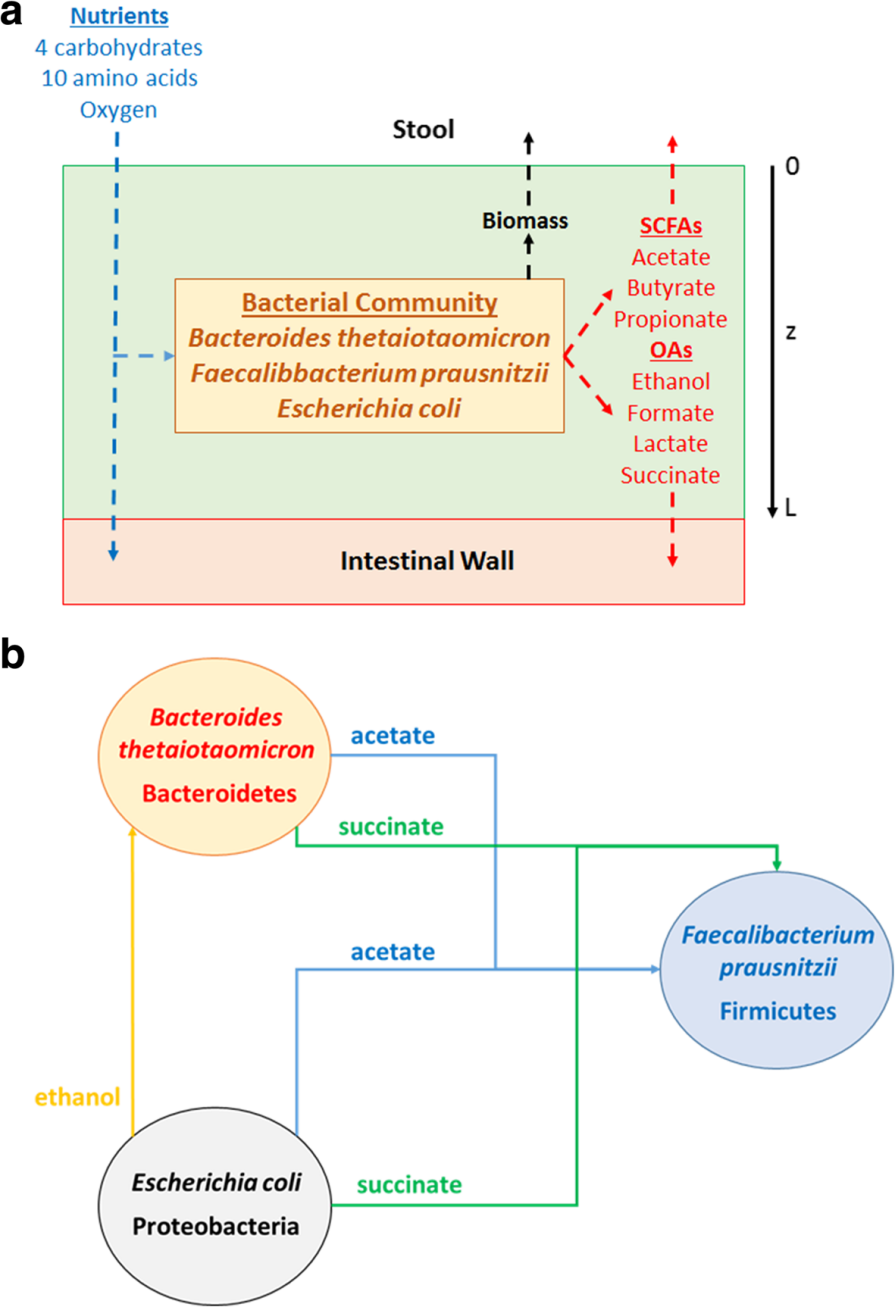
Schematic representation of the *in silico* gut community. **a** The model captured biofilm attachment to the intestinal wall, and diffusion of carbohydrates, amino acids, oxygen, short-chain fatty acids, organic acids and species biomass into and/or out of the biofilm. **b** Modeled cross feeding of byproducts between the three species

The model was formulated assuming nutrients including oxygen diffused into the biofilm at the biofilm-stool boundary and unconsumed nutrients diffused out of the biofilm at the intestine-biofilm boundary (Fig. 1 a). Nutrients could only diffuse unidirectionally from the biofilm because the bulk concentrations in the intestine were assumed to be zero. Short-chain fatty acids (SCFAs) and organic acids (OAs) synthesized by the three bacteria diffused through the biofilm and were removed at both boundaries. Flux balance analysis (FBA) showed that the three species could secrete the SCFAs acetate, propionate and butyrate along with CO_2_ and the OAs ethanol, formate, lactate and succinate. Biomass was assumed to slowly diffuse through biofilm and was removed at the biofilm-stool boundary according to a continuous erosion mechanism [40]. The incorporation of slow biomass diffusion and removal provided a reasonable mechanism to ensure that biomass generation would be balanced by biomass loss such that steady-state solutions could be obtained.

The local extracellular concentration of each nutrient, metabolic byproduct and species biomass was calculated assuming diffusion in the axial direction *z* of the biofilm was the dominant transport mechanism (Fig. 1 a). Therefore, each variable changed as a function of time *t* and space *z* over a fixed biofilm thickness *L*. Local nutrient concentrations were used to calculate local nutrient uptake rates using Michaelis-Menten kinetics. These calculated uptake rates were imposed as lower bounds in FBA linear programs (LPs) for each species to ensure that nutrient transport limitations were honored. LP solution generated the local growth, nutrient uptake and byproduct secretion rates of each species, which served as inputs to partial differential equations (PDEs) describing the local extracellular environment. We incorporated byproduct cross feeding that was shown to enhance community stability in our previous modeling study [41]: acetate and succinate uptake by *F. prausnitzii* and ethanol uptake by *B. thetaiotaomicron* (Fig. 1 b).

The biomass equations for the “strict anaerobes” *B. thetaiotaomicron* and *F. prausnitzii* were formulated assuming oxygen levels were sufficiently low such that growth inhibition was negligible. While *F. prausnitzii* has been shown to remain viable under atmospheric air in the presence of antioxidants [42], *Bacteroides fragilis* has been reported to exhibit inhibited growth at 1 *μ*mol O_2_ [25]. We explored bulk oxygen concentrations up to 5x10 ^−3^ mM, which could generate steady-state oxygen profiles in which 1 *μ*mol was exceeded in the first 3–4 microns of a 40 micron biofilm. Because this region is small and the average oxygen concentration across the biofilm rarely exceeded 100 nM, we concluded the omission of oxygen-mediated growth inhibition was a reasonable simplification. More details on the biofilm model formulation are presented in Additional file 1.

### Model parameterization and solution

Nominal parameter values utilized in the multispecies biofilm model are shown in Table 1. With the exception of the parameters discussed below, the values listed were obtained from our previous study [41]. The nominal bulk oxygen concentration was specified to reflect anaerobic conditions, but *O*_*b*_ values between 0 and 5x10 ^−3^ mM were simulated to predict the effect of oxygen on the three-species community. The oxygen diffusion coefficient was chosen to be within published ranges [43, 44]. The oxygen mass transfer coefficient was specified to achieve a small amount of mass transfer resistance at the stool-biofilm boundary as would be expected for a gas. The carbohydrates arabinose, fructose and galactose were assumed to have the same uptake kinetic parameters as reported for glucose uptake in *E. coli* [45]. For simplicity, all ten amino acids were assumed to have the same uptake kinetic parameters obtained as the average of amino acid dependent values reported for *E. coli* [45]. Oxygen uptake kinetic parameters were obtained from published values reported for *E. coli* [45, 46].

**Table 1:** Nominal parameter values for the multispecies biofilm model

Symbol	Parameter	Value	Units	Source
*L*	Biofilm thickness	40	*μ*m	[44]
*D*_*X*_	Biomass diffusion coefficients	1x10 ^−10^	cm^2^/s	[41]
*k*_*X*_	Biomass mass transfer coefficients	1x10 ^−7^	cm/s	[41]
*X*_*b*_	Biomass bulk concentrations	0	g/L	[41]
*D*_*N*_	Carbohydrate diffusion coefficients	2x10 ^−6^	cm^2^/s	[43]
	Amino acid diffusion coefficients	2x10 ^−6^	cm^2^/s	[43]
	Oxygen diffusion coefficient	8x10 ^−6^	cm^2^/s	[43]
*k*_*N*_	Nutrient mass transfer coefficients	2x10 ^−4^	cm/s	[41]
	Amino acid mass transfer coefficients	2x10 ^−4^	cm/s	[41]
	Oxygen mass transfer coefficient	2x10 ^−2^	cm/s	Specified
*O*_*b*_	Oxygen bulk concentration	0	mM	Specified
*D*_*P*_	Byproduct diffusion coefficients	2x10 ^−6^	cm^2^/s	[43]
*k*_*P*_	Byproduct mass transfer coefficients	5x10 ^−6^	cm/s	[41]
	Butyrate mass transfer coefficient	5x10 ^−5^	cm/s	Tuned
	Propionate mass transfer coefficient	1x10 ^−5^	cm/s	Tuned
*P*_*b*_	Byproduct bulk concentrations	0	mM	[41]
*v*_*max*_	Carbohydrate maximum uptake rates	10	mmol/gDW/h	[45]
	Amino acid maximum uptake rates	1	mmol/gDW/h	[45]
	Oxygen maximum uptake rate	20	mmol/gDW/h	[45]
	Byproduct maximum uptake rates	10	mmol/gDW/h	[41]
*K*_*m*_	Carbohydrate Michaelis-Menten constants	0.5	mM	[45]
	Amino acids Michaelis-Menten constants	0.1	mM	[45]
	Oxygen Michaelis-Menten constant	0.003	mM	[45]
	Byproduct Michaelis-Menten constants	0.5	mM	[41]
*ATPM*	*B. thetaiotaomicron* ATP maintenance	8.43	mmol/gDW/h	[35]
	*F. prausnitzii* ATP maintenance	4.75	mmol/gDW/h	Tuned
	*E. coli* ATP maintenance	5.5	mmol/gDW/h	Tuned

With the remaining parameter values fixed, the biofilm model was tuned to achieve biomass and SCFA fractions within experimental ranges. First the non-growth associated ATP maintenance values within the three genome-scale reconstructions were adjusted to tune the biomass fractions. When all three ATP maintenance values were equal to their published values of 8.43 mmol/gDW/h, the community was unstable and only *B. thetaiotaomicron* was present at steady state due to it superior nutritional efficiency. We did not necessarily expect the published ATP maintenance values to result in coexistence of the three species because our model neglected other phyla (e.g. Actinobacteria), other nutrients (e.g. oligosaccharides) and other species interactions (e.g. Actinobacteria cross feeding of SCFAs and organic acids) as well as host metabolism present in the actual gut environment. Because our goal was to investigate the putative role of oxygen in destabilizing the gut microbiota despite these simplifications, the ATP maintenance values of *F. prausnitzii* and *E. coli* were reduced until an approximate *B. thetaiotaomicron*:*F. prausnitzii*:*E. coli* fraction of 60:30:10 [47] was achieved. Although not discussed here, we found that coexistence with different species fractions was achieved over a range of ATP maintenance values.

With the ATP maintenance values fixed, the three SCFA mass transfer coefficients were adjusted to tune the SCFA fractions. When these mass transfer coefficients were set equal to 5x10 ^−6^ cm/s used in our previous study [41], the acetate:propionate:butyrate fraction was an unrealistic 15:10:75. Therefore, the butyrate and propionate mass transfer coefficients were increased until an approximate fraction of 60:20:20 [13] was obtained. We justified these SCFA dependent values by noting that our model lacks host-microbiota interactions that would strongly affect SCFA levels.

Simulations were performed for three combinations of bulk carbohydrate (CHO) and amino acid concentrations that were chosen to mimic to high CHO, high protein and equal CHO:protein diets. The simulated diets did not include fat because the bacteria are incapable of metabolizing fats. The high CHO diet that served as our nominal case is shown in Table 2, while all three diets are compared in Additional file 1. On a six carbon (C6) basis, the high CHO diet contained 5.0 mM of CHO and 1.5 mM of protein for a total of 6.5 mM of available carbon. The CHO fraction was assumed to consist of 40% glucose and 20% each arabinose, fructose and galactose. The protein fraction was split equally between the 10 amino acids included in the media. The C6 concentrations were corrected for the number of carbons [33] to obtain the actual concentrations used as bulk concentrations in the biofilm model. The bulk concentrations were calculated assuming 1.5 mM of CHO and 5.0 mM of protein for the high protein diet and 3.25 mM of CHO and 3.25 mM of protein for the equal CHO:protein diet.

**Table 2:** Bulk carbohydrate and amino acid concentrations representing a high carbohydrate diet

Nutrient	C6 Concentration	Carbons	Actual concentration
Arabinose	1.000	5	1.200
Fructose	1.000	6	1.000
Galactose	1.000	6	1.000
Glucose	2.000	6	2.000
Total CHO	5.000	–	5.200
Cysteine	0.150	3	0.300
Isoleucine	0.150	6	0.150
Leucine	0.150	6	0.150
Lysine	0.150	6	0.150
Methionine	0.150	5	0.180
Proline	0.150	5	0.180
Serine	0.150	3	0.300
Threonine	0.150	4	0.225
Tryptophan	0.150	11	0.082
Valine	0.150	5	0.180
Total AA	1.500	–	1.897

The biofilm model consisting of a coupled set of nonlinear partial differential equations (PDEs) with embedded linear programs (LPs) was solved by spatially discretizing each PDE into a large set of coupled ordinary differential equations (ODEs) [41, 48]. The resulting ODE system with embedded LPs was solved using the MATLAB code DFBAlab [49]. DFBAlab uses lexicographic optimization to avoid the problem of alternative optima in the LP problems. Following our previous methodology [41], we specified the lexicographic optimization objectives as shown in Additional file 1 with growth maximization being the primary objective. Based on simulations with different numbers of spatial discretization points *N*, we determined that *N*=20 provided a suitable compromise between numerical accuracy and efficiency (Additional file 2). The discretized model consisted of 520 nonlinear ODEs describing the time evolution of the biomass, nutrient and byproduct concentrations at the spatial node points and 1440 LPs for lexicographic optimization. We used Gurobi 6.0 for LP solution, the stiff MATLAB solver ode15s for ODE integration and DFBAlab running in MATLAB 8.5 (R2015a).

Two types of dynamic simulations were performed. The first set of simulations were designed to generate anaerobic initial conditions for the second set of simulations in which the effects of applied oxygen were investigated. An anaerobic simulation for each diet was performed with spatially homogeneous biomass concentrations of 10 g/L for each species as well as spatially homogeneous nutrient and byproduct concentrations. We found that the final steady-state solution was independent of the initial condition. Each applied oxygen simulation was performed by specifying the bulk oxygen concentration and computing the dynamic response from the appropriate anaerobic initial condition.

## Results

### Oxygen induces microbiota dysbiosis

The biofilm metabolic model was tuned for a high CHO diet (Table 2) and anaerobic conditions to generate species abundances (58% *B. thetaiotaomicron*, 30% *F. prausnitzii*, 12% *E. coli*) and SCFA levels (55% acetate, 19% propionate, 26% butyrate) consistent with in vivo studies on the proportions of the modeled phyla (Bacteroidetes, Firmicutes, Proteobacteria) [47] and on SCFA fractions [13]. This anaerobic state was used as the initial condition for biofilm simulations with the bulk oxygen concentration varied from 0 mM (anaerobic) to 5x10 ^−3^ mM (microaerobic).

**Fig. 2:**
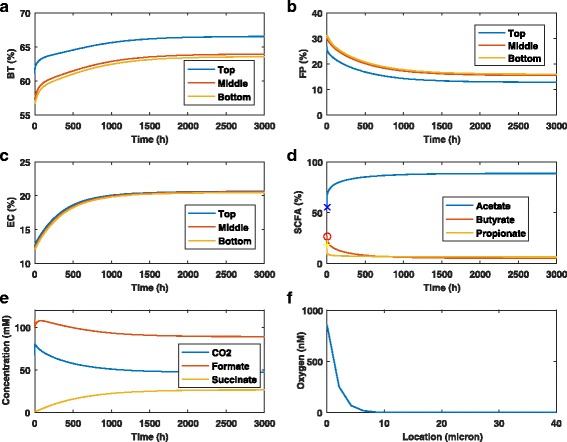
Effect of oxygen on the time evolution of the biofilm community. The bulk oxygen concentration was set at 3x10 ^−3^ mM. **a**–**c** Abundances of *B. thetaiotaomicron*, *F. prausnitzii* and *E. coli* at the top, middle and bottom of the biofilm. **d** SCFA levels averaged across the biofilm where the symbols represent the initial values. **e** Byproduct concentrations averaged across the biofilm. **f** Spatial profile of the steady-state oxygen concentration

Figure 2 shows the time evolution of species abundances, SCFA levels and byproduct concentrations for a single simulation with bulk oxygen concentration of 3x10 ^−3^ mM. *F. prausnitzii* abundances were predicted to decrease across the biofilm by approximately 50% relative to their initial anaerobic values (Fig. 2 b). The lowest abundance was predicted at the top of the biofilm where oxygen was introduced, a particularly unfavorable metabolic niche for *F. prausnitzii*. Predicted *E. coli* abundances increased across the biofilm by 68% relative to their initial anaerobic values (Fig. 2 c). *B. thetaiotaomicron* changes were less severe, with abundances increased by 9–12% depending on the location in the biofilm (Fig. 2 a). The combination of decreased *F. prausnitzii* and increased *E. coli* is consistent with microbiota dysbiosis observed in IBD patients [10, 15] and provides support for the hypothesis that oxygen could be important in IBD pathogenesis.

Due to changes in species abundances, the average acetate level across the biofilm increased by 89% while the propionate and butyrate levels decreased to 6% and 5%, respectively (Fig. 2d). The byproducts formate and CO_2_ secreted by *F. prausnitzii* were predicted to decrease, while succinate used by *F. prausnitzii* as a growth substrate increased sharply (Fig. 2e). The oxygen spatial profile plotted at the final steady state shows that oxygen was present at concentrations greater than 100 nM only in the top 4 microns and never exceeds 900 nM due to limited availability and diffusional restrictions (Fig. 2 f). This result provided some justification for the modeling assumption that oxygen would not substantially inhibit the growth of *B. thetaiotaomicron*.

**Fig. 3:**
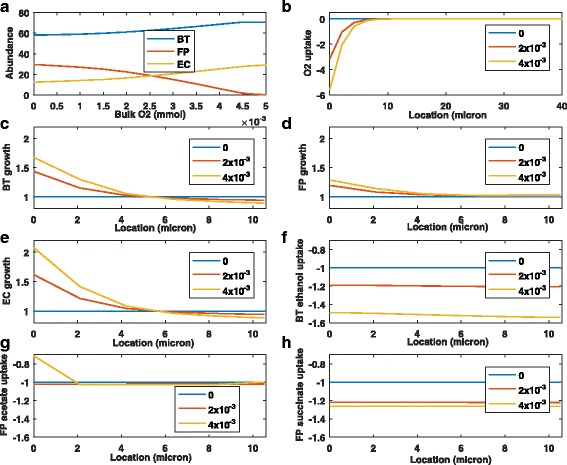
Effect of different oxygen concentrations on the steady-state behavior of the biofilm community. The bulk oxygen concentration was set to a fixed value between 0 and 5x10 ^−3^ mM for each simulation. **a** Species abundances (%) averaged across the biofilm for the range of bulk oxygen concentrations. **b** Species O_2_ uptake rates (mmol/gDW/h) for three bulk oxygen concentrations. **c**-**e** Growth rates (h ^−1^) of *B. thetaiotaomicron*, *F. prausnitzii* and *E. coli* relative to their anaerobic values for three bulk oxygen concentrations. **f**–**h** Uptake rates (mmol/gDW/h) of cross-fed byproducts relative to their anaerobic values for three bulk oxygen concentrations

To gain further insights into the metabolic factors that could contribute to microbiota dysbiosis, we performed simulations for bulk oxygen concentrations from 0 to 5x10 ^−3^ mM. Our model predicted that species abundances were largely insensitive to bulk concentrations less than 1x10 ^−3^ mM (Fig. 3 a), which at steady state produced average biofilm oxygen concentrations less than 20 nM and peak concentrations less than 230 nM. By contrast, the abundances were highly sensitive to bulk concentrations in the range 1x10 ^−3^ to 5x10 ^−3^ mM, which induced average and peak biofilm oxygen concentrations of 30–100 nM and 380–1,370 nM, respectively. Regardless of the bulk concentration, appreciable oxygen uptake occurred only in the top 5 microns of the biofilm (Fig. 3 b).

We examined steady-state growth and uptake fluxes over the top 10 microns of the biofilm for three bulk oxygen concentrations (Fig. 3 c–e). All fluxes were scaled by their anaerobic values at the same spatial location such that the anaerobic profiles were flat lines at unity. This scaling allowed flux increases/decreases relative to the anaerobic case to be directly quantified. At a bulk oxygen concentration of 4x10 ^−3^ mM, *F. prausnitzii* was predicted to achieve modest growth rate enhancements of 20% maximum over the first 5 microns and negligible enhancements over the next 5 microns. By contrast, much larger growth rate enhancements of 200% maximum were predicted for *E. coli* over the first 5 microns with relatively small decreases over the next 5 microns. Similar but less pronounced growth rate profiles were predicted for *B. thetaiotaomicron*, with a maximum enhancement of approximately 70%. These results show that oxygen-mediated growth inhibition is not needed to predict microbiota dysbiosis. Rather oxygen could induce dysbiosis simply by increasing the growth rates of facultative anaerobes and selected “obligate” anaerobes at the expense of less metabolically capable anaerobes such as *F. prausnitzii*.

Given the predicted importance of byproduct cross feeding on community stability [41], we analyzed the ability of the three bacteria to cross feed in the presence of oxygen (Fig. 3 f-h). Predicted *F. prausnitzii* uptake of acetate was largely unaffected by the presence of oxygen, with the exception of reduced uptake in the first two microns at the highest bulk oxygen concentration. While *F. prausnitzii* was predicted to increase succinate uptake with increasing oxygen, *B. thetaiotaomicron* exhibited a larger ethanol uptake increase at the highest oxygen concentration. These results suggest that the inability of *F. prausnitzii* to increase byproduct consumption as efficiently as *B. thetaiotaomicron* might contribute to the dysbiosis.

The ability of *F. prausnitzii* to grow in the presence of oxygen depends on an extracellular electron shuttling mechanism in which flavins and thiols are used for transfering electrons to oxygen [50]. This shuttle is functional only if riboflavin and either cysteine or glutathione are available, which is the case for the healthy gut. Diets rich in riboflavin have been proposed as a means to increase *F. prausnitzii* abundances [51], while Crohn’s disease patients have been shown to have low riboflavin levels [52]. This electron shuttle was included in the *F. prausnitzii* genome-scale reconstruction [34], and our simulated gut environment that included both riboflavin and cysteine allowed the shuttle to be active. To examine the effect of removing this shuttle, we constrained the upper bound of the FLVXre reaction in the reconstruction [34] to zero. While removal of the shuttle accelerated the effect of oxygen on dysbiosis, the effect was not dramatic (Additional file 2). This result suggests that unmodeled oxygen toxicity could play a role in *F. prausnitzii* decline and IBD pathogenesis.

### Oxygen sensitivity depends on diet

While diet has been hypothesized to be a key factor in IBD pathogenesis, the impact of different dietary components on disease progression and recurrence are not well understood [7, 10]. Many alternative diets have been proposed based on clinical heuristics, but there remains no standard clinical dietary recommendations for IBD patients [53]. Some studies have shown that diets rich in readily fermentable monosaccharides and oligosaccharides are risk factors for IBD development [53]. These studies have motivated the development of the excluded fermentable oligo-, di-, monosaccharides and polyols (FOMAP) diet for IBD patients. On the other hand, the western diet rich in animal protein also has been correlated with IBD progression [53, 54].

To investigate the possible impact of dietary carbohydrates and proteins on microbiota dysbiosis in the presence of oxygen, we performed biofilm simulations with three *in silico* diets: high carbohydrate (CHO), high protein (PRO) and equal CHO:PRO. The three diets are listed in Additional file 1 with respect to the bulk concentrations of the different nutrients. To allow a fair comparison, the three diets had equal carbon content on a C6 basis: high CHO (5.0 mM CHO, 1.5 mM PRO), high PRO (1.5 mM CHO, 5.0 mM PRO), and equal CHO:PRO (3.25 mM CHO, 3.25 mM PRO).

**Fig. 4:**
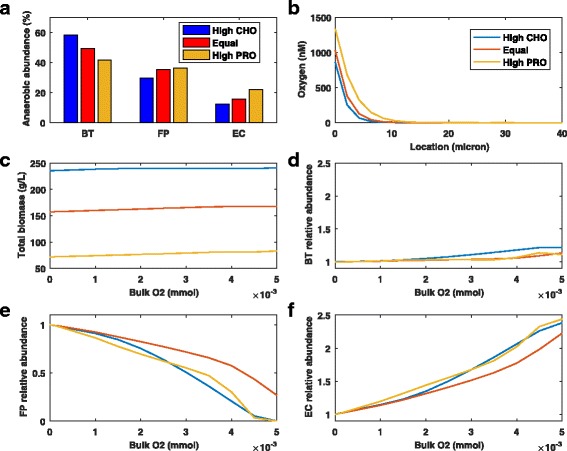
Combined effects of different diets and oxygen concentrations on the steady-state abundances of the three species. Bulk carbohydrate and amino acid concentrations were varied according to the diet and the bulk oxygen concentration was set to a fixed value between 0 and 5x10 ^−3^ mM. **a** Anaerobic species abundances averaged across the biofilm. **b** Spatial profiles of the steady-state oxygen concentration. **c** Total biomass produced by the three species averaged across the biofilm. **d**–**f** Species abundances relative to anaerobic values averaged across the biofilm

Different diets were predicted to have a large impact on species abundances under anaerobic conditions (Fig. 4 a) with high CHO favoring *B. thetaiotaomicron* and high PRO favoring *F. prausnitzii* and *E. coli*, presumably due to the efficient CHO metabolism of *B. thetaiotaomicron* [33]. When compared at a single bulk oxygen concentration of 3x10 ^−3^ mM, spatial profiles of the steady-state oxygen concentration (Fig. 4 b) showed that the high PRO diet allowed the deepest penetration into the biofilm due to decreased oxygen utilization. As expected, the high CHO diet was predicted to accumulate the highest total biomass from the three species owing to carbohydrates being a better energy source than amino acids.

Because the diets produced different anaerobic species abundances, we scaled the abundances at each bulk oxygen concentration by the anaerobic values for that diet such that all anaerobic abundances were unity (Fig. 4 c–e). This scaling allowed the impact of oxygen for the three diets to be directly compared. The diets were predicted to have little differential effect on *B. thetaiotaomicron* abundances, with increases of 11–21% observed over the entire oxygen range. Interestingly, the equal CHO-PRO diet was predicted to most effectively delay the onset of dysbiosis with increasing oxygen. At the highest bulk oxygen concentration of 5x10 ^−3^ mM, the *F. prausnitzii* abundance remained at 27% of its anaerobic value compared to less than 0.2% for the other two diets. The equal CHO-PRO diet slightly slowed *E. coli* expansion compared to the other diets, allowing *F. prausnitzii* to co-exist at higher oxygen levels. These simulations support the provocative hypothesis that a balanced diet of carbohydrates and protein would most effectively delay IBD pathogenesis.

**Fig. 5:**
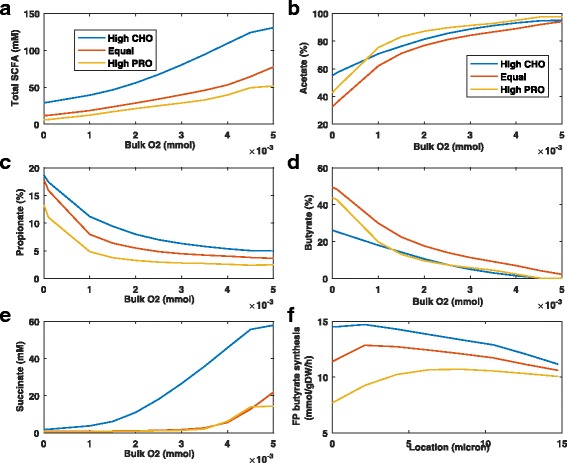
Combined effects of different diets and oxygen concentrations on the steady-state levels of SCFAs and succinate. Bulk carbohydrate and amino acid concentrations were varied according to the diet and the bulk oxygen concentration was set to a fixed value between 0 and 5x10 ^−3^ mM. **a** Total SCFA concentration averaged across the biofilm. **b**–**d** Acetate, propionate and butyrate levels averaged across the biofilm. **e** Succinate concentration averaged across the biofilm. **f** Spatial profile of the *F. prausnitzii* butyrate synthesis rate at a bulk oxygen concentration of 3x10 ^−3^ mM

We also examined the combined effects of diet and oxygen on SCFA and succinate levels. For all three diets, the total SCFA concentration was predicted to increase with increasing bulk oxygen concentration (Fig. 5 a). The high CHO diet produced the highest SCFA concentrations, as would be expected due to higher carbohydrate availability [14]. Both diet and oxygen were predicted to have dramatic effects on the split between the three SCFAs. Under anaerobic conditions, the acetate level decreased with increasing amino acid content while the propionate level was substantially lower for the high protein diet and the butyrate level was substantially lower for the high CHO diet (Fig. 5 b–d). These trends were consistent with those observed for the species abundances under anaerobic conditions (Fig. 4). The acetate level dramatically increased while the propionate and butyrate levels sharply decreased with increasing oxygen for all diets.

Higher butyrate levels were predicted for the equal CHO-PRO diet due to higher *F. prausnitzii* abundances (see Fig. 4 d). Steady-state spatial profiles of the *F. prausnitzii* butyrate synthesis rate at a bulk oxygen concentration of 3x10 ^−3^ mM showed that enhanced buytrate levels predicted for the equal CHO-PRO diet were attributable to higher *F. prausnitzii* abundances not higher butyrate synthesis (Fig. 5 f). The succinate concentration increased rapidly with increasing oxygen (Fig. 5 e) due to the decreasing *F. prausnitzii* abundance and was particularly high for the CHO diet due to enhanced succinate synthesis by *B. thetaiotaomicron*. Collectively these predictions suggest that buytrate levels are mainly determined by biofilm oxygen concentrations rather than dietary components.

### Feedback between microbiota and host can amplify the oxygen effect

A central tenet of the oxygen hypothesis is that microbiota dysbiosis induces inflammation in the host, which causes increased release of oxygen and reactive oxygen species into the intestinal lumen thereby amplifying the dysbiosis process. This positive feedback mechanism would be expected to increase IBD pathogenesis compared to a one way interaction where oxygen release causes dysbiosis but dysbiosis does not affect oxygen levels. The simulations presented thus far modeled this one way interaction by fixing the bulk oxygen concentration and predicting the species abundances.

**Fig. 6:**
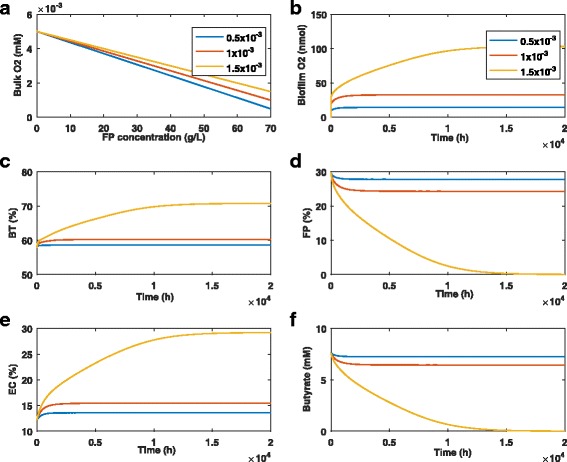
Effect of oxygen perturbations with host-microbiota feedback. The oxygen perturbation was set to one value in the legend at time zero and sustained throughout the simulation. **a** Linear relationship mimicking the putative host-microbiota feedback mechanism. **b** Biofilm oxygen concentration averaged across the biofilm. **c**–**e** Species abundances averaged across the biofilm. **f** Butyrate concentration averaged across the biofilm

To mimic positive feedback between the microbiota and host, we developed a simple phenomenological relation between butyrate producing *F. prausnitzii* and the bulk oxygen concentration (see Additional file 1). The linear relationship was based on the assumption that a sustained oxygen perturbation occurred at time zero when the *F. prausnitzii* biomass concentration was at its anaerobic value (Fig. 6 a). From this point the bulk oxygen concentration increased linearly until the *F. prausnitzii* biomass concentration was zero, at which point the bulk oxygen concentration was assumed to be 5x10 ^−3^ mM. This phenomenological relation was consistent with a recent experimental study demonstrating a mechanistic link between reduced butyrate levels and increased oxygen levels [26]. While the incorporation of positive feedback can produce bistability and hysteresis [55], we observed no evidence of these behaviors. This result appears to be consistent with the available experimental literature, which has not yet demonstrated the existence of switching behavior.

As observed for a fixed bulk oxygen concentration (see Fig. 3), small perturbations of 0.5x10 ^−3^ mM and 1x10 ^−3^ mM were predicted to induce only small changes in the species abundances (Fig. 6 c–e) and the butyrate concentration (Fig. 6 f). For these two cases, the biofilm oxygen concentration was maintained below 35 nM (Fig. 6 b) and dysbiosis was not substantially amplified. However, a slightly larger oxygen perturbation of 1.5x10 ^−3^ mM was predicted to eliminate *F. prausnitzii* and butyrate entirely with the *B. thetaiotaomicron* and *E. coli* abundances increased by 22% and 138%, respectively. This perturbation resulted in a much higher oxygen level of ∼100 nM in the biofilm. When compared with the same perturbation for the fixed bulk oxygen case (see Fig. 3), the positive feedback mechanism produced much more severe dysbiosis. Interestingly, the timescale of the dysbiosis dynamics with host feedback was on the order of 20,000 hours (2.3 years) while that for the fixed bulk oxygen case was about 3,000 hours (4.1 months). Hence, our model predicted that bidirectional host-microbiota feedback will substantially amplify microbiota dysbiosis but greatly lengthen the dysbiosis timescale.

Antibiotics such as ciprofloxacin and metronidazole often are used a a first-line therapy for IBD patients [7]. While early studies with small patient numbers seemed to provide some support for this practice, more recent studies with much larger patient populations and omics analyses have cast doubt on the efficacy of antibiotic treatment [29], especially for Crohn’s disease [56, 57]. In fact, these studies suggest that antibiotics actually could be detrimental by reducing microbial diversity, decreasing the abundances of beneficial species and reducing colonization resistance to pathogenic bacteria. To investigate the possible effect of antibiotic treatment *in silico*, we introduced a simple modification to our biofilm model to account for antibiotic mediated cell death (see Additional file 1). The antibiotic level was held constant throughout the biofilm at a specified concentration assuming the release rate of antibiotic from a cell was much faster than antibiotic diffusional dynamics. The host-microbiota feedback relationship was included without an oxygen perturbation such that increased oxygen levels only could result from decreases in *F. prausnitzii* concentration. Simulation results were qualitatively similar to those obtained for oxygen perturbations, with a sufficiently large antibiotic level inducing high biofilm oxygen concentrations and essentially eliminating *F. prausnitzii* (see Additional file 2). These predictions provide some support for the hypothesis that antibiotic treatment could exasperate IBD pathogenesis.

## Discussion

Ulcerative colitis and Crohn’s disease are highly complex inflammatory bowel diseases (IBDs) that involve myriad interactions between the gut microbiota, host immune system and diverse environmental factors such as diet and antibiotics. Our current understanding of these diseases is incomplete, which limits the development of effective and generally applicable therapies. Future advancements will depend on careful dissection of host-microbiota-environmental interactions in suitable animal models and actual IBD patients. Because the gut microbiota are known to play a critical role in IBD pathogenesis, improved understanding of the complex interactions between microbial species in healthy and diseased gut environments is a prerequisite to gaining a holistic picture of IBD.

The premise of the current study was that insights into microbiota dysbiosis could be gained through the development and analysis of a multispecies metabolic model. With regard to species diversity, we formulated a minimal model [41] with a representative species from the two dominant phyla in the healthy gut (the obligate anaerobes *Faecalibacterium prausnitzii* from the phylum Firmicutes and *Bacteroides thetaiotaomicron* from the phylum Bacteroidetes) and one species known to be overrepresented in the IBD gut (the facultative anaerobe *Escherichia coli* from the phylum Proteobacteria). However, the model incorporated detailed descriptions of species metabolism through genome-scale metabolic reconstructions and of species interactions through nutrient competition and byproduct cross feeding. We believe that our bottoms-up modeling philosophy meshes well with top-down modeling approaches based on more complex gut communities but simpler descriptions of intra- and intercellular metabolism [58–60].

Our biofilm metabolic model was designed to test the hypothesis that oxygen plays a key role in IBD microbiota dysbiosis by increasing the abundance of facultative anaerobes at the expense of obligate anaerobes [10, 15]. The model was well suited for this purpose since dysbiosis has been strongly correlated with decreasing *F. prausnitzii* and increasing *E. coli* densities, while no clear trend has been established for *B. thetaiotaomicron* [15]. We found that dysbiosis consistent with IBD disease progression could be qualitatively predicted solely based on metabolic differences between the three species. At lumen oxygen concentrations between 1x10 ^−3^ mM and 5x10 ^−3^ mM, our biofilm model predicted that the *F. prausnitzii* population would be rapidly eliminated due to its inability to efficiently utilize oxygen for growth enhancement. Over the same oxygen range, the *E. coli* abundance was predicted to increase almost 140% compared to its anaerobic value. *B. thetaiotaomicron* was predicted to exhibit a much smaller increase of about 20%, which was consistent with some experimental investigations [15]. *B. thetaiotaomicron* decline observed in some other studies [15] may be attributable to oxygen inhibition of growth, which have been reported to occur around 1,000 nM for the closely related gut bacterium *B. fragilis* [25]. Our model did not include this effect because the average oxygen concentration across the biofilm rarely exceeded 100 nM, although the peak oxygen concentration in the first 3–4 microns could exceed 1,000 nM. Oxygen toxicity is an interesting topic for future experimental and modeling studies.

We simulated three nutrient environments that differed with respect to carbohydrate and amino acid (protein) content to explore the effect of diet on microbiota dysbiosis: high carbohydrate (CHO), high protein (PRO) and equal CHO-PRO. We expected the high protein diet to provide the most benefit to *F. prausnitzii* due to the lack of carbohydrates to support *B. thetaiotaomicron* and *E. coli* growth. Interestingly, the equal CHO-PRO diet allowed *F. prausnitzii* to most effectively compete metabolically with the other two bacteria over a range of oxygen concentrations. Based on anaerobic abundances, the equal CHO-PRO diet seemed to represent a “sweet spot” for *F. prausnitzii*. Higher carbohydrate content favored *B. thetaiotaomicron*, while higher protein content favored *E. coli*. These predictions appeared to be consistent with clinical studies showing that diets rich in fermentable monosaccharides/oligosaccharides [53] or rich in animal protein [54] are risk factors for IBD development. To limit model complexity, the simulated gut nutrients were restricted to four monosaccharides and ten amino acids expected to be available for consumption by gut bacteria. Future inclusion of the other ten amino acids and more complex carbohydrates such as oligo- and polysaccharides would expand usefulness of the model but substantially increase computational demand since carbohydrate breakdown by *B. thetaiotaomicron* is modeled as an extracellular process [33] and would require the inclusion of additional partial differential equations.

The previous simulations were performed by setting the lumen oxygen concentration to a constant value and then predicting the effect on the microbiota. While informative, this approach neglected positive feedback between microbiota dysbiosis and host inflammation, which is believed to be the cause for putative oxygen increases [19]. As a non-mechanistic means to investigate this feedback mechanism, we incorporated a simple linear relation between the *F. prausnitzii* concentration and the lumen oxygen concentration such that an oxygen perturbation would induce *F. prausnitzii* decline, which would trigger increasing oxygen levels. Host-microbiota feedback did not substantially change the range of lumen oxygen concentrations where dysbiosis was predicted, but the transition from normal species abundances to severe dysbiosis was much more dramatic and occurred over a much longer timescale. These results demonstrate how IBD might progress over several years with few noticeable effects and then suddenly start producing severe disease symptoms. Similar predictions were obtained with sustained antibiotic treatment replacing a sustained oxygen perturbation, consistent with recent clinical studies questioning the use of antibiotics as a first-line therapy for IBD patients [29, 57]. Clearly modeling of host-microbiota feedback would be improved by incorporating a mechanistic model of host metabolism, which would be possible for the mouse by using a genome-scale metabolic reconstruction of *Mus musculus* [33] to add a fourth species to the community. This extension is an exciting target for future modeling research and could allow more direct testing of model predictions in gnotobiotic mice.

While we believe that the gut community model developed in this study yielded valuable insights into the possible role of oxygen in IBD pathogenesis, numerous improvements can be envisioned to improve model fidelity and usefulness. In addition to previous comments about expanding the modeled nutrients and incorporating host metabolism, the model could be expanded to include additional gut species for which curated genome-scale metabolic reconstructions are available [61–64]. This extension would allow the incorporation of more complex cross feeding relationships and investigation of their effects on community robustness in healthy and diseased states. We modeled the three species community as a biofilm due to experimental studies that provide substantial support for the hypothesis that gut microbes organize into spatially structured multispecies biofilms [65–67]. The addition of planktonic populations in the get lumen that compete for nutrients and cross feed byproducts with biofilm communities associated with the host mucosa would provide a more accurate picture of species interactions. A drawback of our bottoms-up modeling approach is the need for parameters such as species specific nutrient uptake kinetics and metabolite dependent mass transfer coefficients. While currently unknown for most gut species, many of these parameters could be determined from carefully designed planktonic and biofilm experiments with the individual species.

## Conclusions

Inflammatory bowel diseases (IBD) involve dysbiosis of the commensal gut microbiota characterized by a significant reduction of obligate anaerobes and a sharp increase in facultative anaerobes. We developed a multispecies biofilm metabolic model to test the hypothesis that IBD dysbiosis is mediated by increased oxygen levels in the gut. The biofilm model was built upon genome-scale metabolic reconstructions of representative species from the three dominant phyla in the human gut: *Bacteroides thetaiotaomicron*, an obligate anaerobe from the phylum Bacteroidetes that secretes the short-chain fatty acids (SCFAs) acetate and propionate; *Faecalibacterium prausnitzii*, an obligate anaerobe from the phylum Firmicutes that secretes the health-promoting SCFA butyrate; and *Escherichia coli*, a facultative anaerobe from the phylum Proteobacteria present at low levels in healthy individuals. The metabolic reconstructions were combined with reaction-diffusion transport equations and uptake kinetics for key nutrients and putative cross-fed byproducts. By specifying bulk concentrations of nutrients including oxygen at the boundary of the simulated biofilm, the model was able to predict the impact of metabolite concentration gradients on multispecies metabolism as a function of time and location.

The key predictions of our biofilm metabolic modeling study were:

1. Bulk oxygen concentrations as low as 1 micromolar were sufficient to qualitatively reproduce experimental data showing a sharp decrease of *F. prausnitzii* and a large increase of *E. coli*, demonstrating that dysbiosis consistent with IBD disease progression could be predicted solely based on metabolic differences between key species.
2. Similar predictions were obtained with sustained antibiotic treatment replacing a sustained oxygen perturbation, consistent with clinical studies linking broad spectrum antibiotics and IBD.
3. Oxygen sensitivity was dependent on diet with balanced carbohydrate and protein content predicted to increase the oxygen range over which *F. prausnitzii* could remain competitive and the butyrate-depleting effect of IBD could be sublimated.
4. Host-microbiota feedback included through a simple linear mechanism caused dysbiosis to occur over a long timescale of months, indicating how IBD might progress slowly with few noticeable effects and then suddenly start producing severe disease symptoms.

We believe that our model predictions could be tested through the development of an appropriate in vitro system of the three species community and testing of microbiota-host interactions in gnotobiotic mice.

## Additional files

Additional file 1 Pdf file for biofilm model description. (PDF 179 kb)

Additional file 2 Pdf file for other simulation results. (PDF 181 kb)

Additional file 3 Zip folder containing all the MATLAB codes used for this paper. (ZIP 447 kb)

## Abbreviations

CHO: Carbohydrates
DFBAlab: Dynamic flux balance analysis laboratory
FBA: Flux balance analysis
FOMAP: Fermentable oligo-,di-,monosaccharides and polyols
IBD: Inflammatory bowel diseases
LP: Linear programming
OA: Organic acid
ODE: Ordinary differential equation
PDE: Partial differential equation
PRO: Protein
SCFA: Short chain fatty acid

## References

[1] Danese S, Fiocchi C (2011). Ulcerative colitis. New England J Med.

[2] Baumgart DC, Sandborn WJ (2012). Crohn’s disease. Lancet.

[3] Colombel JF, Mahadevan U (2017). Inflammatory bowel disease 2017: Innovations and changing paradigms. Gastroenterology.

[4] Bernstein CN (2015). Treatment of IBD: where we are and where we are going. Am J Gastroenterol.

[5] Lewis RT, Maron DJ (2010). Efficacy and complications of surgery for Crohn’s disease. Gastroenterol Hepatol.

[6] Mehta F (2016). Report: economic implications of inflammatory bowel disease and its management. Am J Manag Care.

[7] Hold GL, Smith M, Grange C, Watt ER, El-Omar EM, Mukhopadhya I (2014). Role of the gut microbiota in inflammatory bowel disease pathogenesis: what have we learnt in the past 10 years?. World J Gastroenterol: WJG.

[8] Thompson JA, Oliveira RA, Xavier KB (2016). Chemical conversations in the gut microbiota. Gut Microbes.

[9] Honda K, Littman DR (2016). The microbiota in adaptive immune homeostasis and disease. Nature.

[10] DeGruttola AK, Low D, Mizoguchi A, Mizoguchi E (2016). Current understanding of dysbiosis in disease in human and animal models. Inflamm Bowel Dis.

[11] Shreiner AB, Kao JY, Young VB (2015). The gut microbiome in health and in disease. Curr Opin Gastroenterol.

[12] Theriot CM, Young VB (2015). Interactions between the gastrointestinal microbiome and *Clostridium difficile*. Annu Rev Microbiol.

[13] Byrne C, Chambers E, Morrison D, Frost G (2015). The role of short chain fatty acids in appetite regulation and energy homeostasis. Int J Obes.

[14] den Besten G, van Eunen K, Groen AK, Venema K, Reijngoud DJ, Bakker BM (2013). The role of short-chain fatty acids in the interplay between diet, gut microbiota, and host energy metabolism. J lipid Res.

[15] Matsuoka K, Kanai T (2015). The gut microbiota and inflammatory bowel disease. Seminars in Immunopathology. vol. 37, No.1.

[16] Lu C, Zhao X, Lai MA, Lopez-Yglesias AH, Quarles EK, Lo C, Smith KD. Commensal *E. coli* induced colonization resistance against mucosal *Salmonella* infection. Am Assoc Immnol. 2016;:66–11.

[17] Ahmed I, Roy BC, Khan SA, Septer S, Umar S (2016). Microbiome, metabolome and inflammatory bowel disease. Microorganisms.

[18] Øyri SF, Műzes G, Sipos F (2015). Dysbiotic gut microbiome: A key element of Crohn’s disease. Comp Immunol Microbiol Infect Dis.

[19] Rigottier-Gois L (2013). Dysbiosis in inflammatory bowel diseases: the oxygen hypothesis. ISME J.

[20] Zhu H, Li YR (2012). Oxidative stress and redox signaling mechanisms of inflammatory bowel disease: updated experimental and clinical evidence. Exp Biol Med.

[21] Albenberg L, Esipova TV, Judge CP, Bittinger K, Chen J, Laughlin A, Grunberg S, Baldassano RN, Lewis JD, Li H (2014). Correlation between intraluminal oxygen gradient and radial partitioning of intestinal microbiota. Gastroenterology.

[22] Hartman AL, Lough DM, Barupal DK, Fiehn O, Fishbein T, Zasloff M, Eisen JA (2009). Human gut microbiome adopts an alternative state following small bowel transplantation. Proc Natl Acad Sci.

[23] Kelly CJ, Zheng L, Campbell EL, Saeedi B, Scholz CC, Bayless AJ, Wilson KE, Glover LE, Kominsky DJ, Magnuson A (2015). Crosstalk between microbiota-derived short-chain fatty acids and intestinal epithelial HIF augments tissue barrier function. Cell Host Microbe.

[24] Miquel S, Leclerc M, Martin R, Chain F, Lenoir M, Raguideau S, Hudault S, Bridonneau C, Northen T, Bowen B (2015). Identification of metabolic signatures linked to anti-inflammatory effects of *Faecalibacterium prausnitzii*. MBio.

[25] Baughn AD, Malamy MH (2004). The strict anaerobe *Bacteroides fragilis* grows in and benefits from nanomolar concentrations of oxygen. Nature.

[26] Byndloss MX, Olsan EE, Rivera-Chávez F, Tiffany CR, Cevallos SA, Lokken KL, Torres TP, Byndloss AJ, Faber F, Gao Y (2017). Microbiota-activated ppar- *γ* signaling inhibits dysbiotic enterobacteriaceae expansion. Science.

[27] De Vos WM (2015). Microbial biofilms and the human intestinal microbiome. npj Biofilms Microbiomes.

[28] Macfarlane S, Bahrami B, Macfarlane GT (2011). Mucosal biofilm communities in the human intestinal tract. Adv Appl Microbiol.

[29] von Rosenvinge EC, O’May GA, Macfarlane S, Macfarlane GT, Shirtliff ME (2013). Microbial biofilms and gastrointestinal diseases. Pathog Dis.

[30] Greenblum S, Turnbaugh PJ, Borenstein E (2012). Metagenomic systems biology of the human gut microbiome reveals topological shifts associated with obesity and inflammatory bowel disease. Proc Natl Acad Sci.

[31] Levy R, Borenstein E (2013). Metabolic modeling of species interaction in the human microbiome elucidates community-level assembly rules. Proc Natl Acad Sci.

[32] Henson MA, Hanly TJ (2014). Dynamic flux balance analysis for synthetic microbial communities. IET Syst Biol.

[33] Heinken A, Sahoo S, Fleming RM, Thiele I (2013). Systems-level characterization of a host-microbe metabolic symbiosis in the mammalian gut. Gut microbes.

[34] Heinken A, Khan MT, Paglia G, Rodionov DA, Harmsen HJ, Thiele I (2014). Functional metabolic map of *Faecalibacterium prausnitzii*, a beneficial human gut microbe. J Bacteriol.

[35] Baumler DJ, Peplinski RG, Reed JL, Glasner JD, Perna NT (2011). The evolution of metabolic networks of *E. coli*. BMC Syst Biol.

[36] Flint HJ, Scott KP, Duncan SH, Louis P, Forano E (2012). Microbial degradation of complex carbohydrates in the gut. Gut microbes.

[37] Zhao L (2013). The gut microbiota and obesity: from correlation to causality. Nat Rev Microbiol.

[38] Dai ZL, Wu G, Zhu WY (2011). Amino acid metabolism in intestinal bacteria: links between gut ecology and host health. Front Biosci.

[39] Larocque M, Chénard T, Najmanovich R (2014). A curated *C. difficile* strain 630 metabolic network: prediction of essential targets and inhibitors. BMC Syst Biol.

[40] Horn H, Lackner S. Modeling of biofilm systems: a review. In: Productive Biofilms. Springer International Publishing: 2014. p. 53–76. 10.1007/10_2014_275 25163572

[41] Henson MA, Phalak P (2017). Byproduct cross feeding and community stability in an in silico biofilm model of the gut microbiome. Processes.

[42] Khan MT, van Dijl JM, Harmsen HJ (2014). Antioxidants keep the potentially probiotic but highly oxygen-sensitive human gut bacterium *Faecalibacterium prausnitzii* alive at ambient air. PloS ONE.

[43] Stewart PS (2003). Diffusion in biofilms. J Bacteriol.

[44] Stewart PS (1998). A review of experimental measurements of effective diffusive permeabilities and effective diffusion coefficients in biofilms. Biotech Bioeng.

[45] Meadows AL, Karnik R, Lam H, Forestell S, Snedecor B (2010). Application of dynamic flux balance analysis to an industrial *Escherichia coli* fermentation. Metab Eng.

[46] Carlson R, Srienc F (2004). Fundamental *Escherichia coli* biochemical pathways for biomass and energy production: identification of reactions. Biotech Bioeng.

[47] Spor A, Koren O, Ley R (2011). Unravelling the effects of the environment and host genotype on the gut microbiome. Nat Rev Microbiol.

[48] Phalak P, Chen J, Carlson RP, Henson MA (2016). Metabolic modeling of a chronic wound biofilm consortium predicts spatial partitioning of bacterial species. BMC Syst Biol.

[49] Gomez JA, Hoffner K, Barton PI (2014). DFBAlab: a fast and reliable MATLAB code for dynamic flux balance analysis. BMC Bioinforma.

[50] Khan MT, Duncan SH, Stams AJ, Van Dijl JM, Flint HJ, Harmsen HJ (2012). The gut anaerobe *Faecalibacterium prausnitzii* uses an extracellular electron shuttle to grow at oxic–anoxic interphases. ISME J.

[51] Khan MT, Browne WR, van Dijl JM, Harmsen HJ. How can *Faecalibacterium prausnitzii* employ riboflavin for extracellular electron transfer? 2012:1433–1440. 10.1089/ars.2012.4701 22607129

[52] Kuroki F, Iida M, Tominaga M, Matsumoto T, Hirakawa K, Sugiyama S, Fujishima M (1993). Multiple vitamin status in Crohn’s disease. Dig Dis Sci.

[53] Dixon LJ, Kabi A, Nickerson KP, McDonald C (2015). Combinatorial effects of diet and genetics on inflammatory bowel disease pathogenesis. Inflamm Bowel Dis.

[54] Haskey N, Gibson DL (2017). An examination of diet for the maintenance of remission in inflammatory bowel disease. Nutrients.

[55] Ozbudak EM, Thattai M, Lim HN, Shraiman BI, Van Oudenaarden A (2004). Multistability in the lactose utilization network of escherichia coli. Nature.

[56] Card T, Logan R, Rodrigues L, Wheeler J (2004). Antibiotic use and the development of Crohn’s disease. Gut.

[57] Gevers D, Kugathasan S, Denson LA, Vázquez-Baeza Y, Van Treuren W, Ren B, Schwager E, Knights D, Song SJ, Yassour M (2014). The treatment-naive microbiome in new-onset Crohn’s disease. Cell Host Microbe.

[58] Van Hoek MJ, Merks RM (2017). Emergence of microbial diversity due to cross-feeding interactions in a spatial model of gut microbial metabolism. BMC Syst Biol.

[59] Kettle H, Louis P, Holtrop G, Duncan SH, Flint HJ (2015). Modelling the emergent dynamics and major metabolites of the human colonic microbiota. Environ Microbiol.

[60] Muñoz-Tamayo R, Laroche B, Walter É, Doré J, Leclerc M (2010). Mathematical modelling of carbohydrate degradation by human colonic microbiota. J Theor Biol.

[61] Heinken A, Thiele I (2015). Systematic prediction of health-relevant human-microbial co-metabolism through a computational framework. Gut Microbes.

[62] Ji B, Nielsen J. From next-generation sequencing to systematic modeling of the gut microbiome. Front Genet. 2015; 6. 10.3389/fgene.2015.00219 PMC4477173 26157455

[63] Shoaie S, Nielsen J. Elucidating the interactions between the human gut microbiota and its host through metabolic modeling. Front Genet. 2014; 5. 10.3389/fgene.2014.00086 PMC4000997 24795748

[64] Thiele I, Heinken A, Fleming RM (2013). A systems biology approach to studying the role of microbes in human health. Curr Opin Biotechnol.

[65] Donelli G, Vuotto C, Cardines R, Mastrantonio P (2012). Biofilm-growing intestinal anaerobic bacteria. FEMS Immunol Med Microbiol.

[66] Macfarlane S, Dillon J (2007). Microbial biofilms in the human gastrointestinal tract. J Appl Microbiol.

[67] Ouwerkerk JP, de Vos WM, Belzer C (2013). Glycobiome: bacteria and mucus at the epithelial interface. Best Practice Res Clinical Gastroenterol.

