# Supplementary material for: Microbiota dysbiosis in inflammatory bowel diseases: in silico investigation of the oxygen hypothesis
Source: BMC Syst Biol. 2017 Dec 28;11:145. doi: 10.1186/s12918-017-0522-1 (PMC5745886; doi:10.1186/s12918-017-0522-1)
Supplement: Supplementary file 1 — Pdf file for biofilm model description. (PDF 179 kb) [file 12918_2017_522_MOESM1_ESM.pdf]

Additional file 1 — Biofilm Model Description

Model equations

The multispecies biofilm model was based on several simplifying assumptions including: (1) cell death was negligible compared to cell growth; (2) the inhibitory effects of organic acids were insufficient to strongly affect species metabolism; and (3) host circadian rhythms were neglected by predicting the time-average behavior at long times rather than daily variations. Under these assumptions, the biomass equation for each species had the form,

$$\frac{\partial X_i}{\partial t} = \mu_i X_i + D_{X,i}\frac{\partial^2 X_i}{\partial z^2}, \tag{1}$$

where $X_i(z,t)$ is the local biomass concentration (g/L) of species i, $\mu_i(z,t)$ is the local growth rate (h^{-1}) calculated from the metabolic reconstruction and $D_{X,i}$ is a biomass diffusion coefficient. Boundary conditions were imposed to represent zero biomass flux at the intestine-biofilm boundary ($z = L$) and biomass removal by continuous erosion [1] at the biofilm-stool interface ($z = 0$),

$$\frac{\partial X_i(L,t)}{\partial z} = 0, \qquad -D_{X,i}\frac{\partial X_i(0,t)}{\partial z} = k_{X,i}[X_{b,i}(0) - X_i(0,t)], \tag{2}$$

where $k_{X,i}$ is a biomass mass transfer coefficient and $X_{b,i}(0)$ is the bulk planktonic concentration of species i in the stool, which was assumed to be zero for simplicity. The nutrient transport equations had the form,

$$\frac{\partial N_i}{\partial t} = \sum_{j=1}^{n} v_{i,j} X_j + D_{N,i}\frac{\partial^2 N_i}{\partial z^2}, \tag{3}$$

where $N_i(z,t)$ is the local concentration of nutrient i (arabinose, fructose, galactose, glucose, oxygen, cysteine, isoleucine, leucine, lysine, methionine, proline serine, threonine, tryptophan, valine), $v_{i,j}(z,t)$ is the uptake flux (mmol/gDW/h) of nutrient i by species j calculated from the metabolic reconstruction, $D_{N,i}$ is a nutrient diffusion coefficient and $n = 3$ is the number of species. Boundary conditions were imposed such that unconsumed nutrients could be removed from either boundary,

$$-D_{N,i}\frac{\partial N_i(0,t)}{\partial z} = k_{N,i}[N_{b,i}(0) - N_i(0,t)], \qquad -D_{N,i}\frac{\partial N_i(L,t)}{\partial z} = k_{N,i}[N_{b,i}(L) - N_i(L,t)], \tag{4}$$

where $k_{N,i}$ is a nutrient mass transfer coefficient, and $N_{b,i}(0)$ and $N_{b,i}(L)$ are bulk concentrations of nutrient i. The bulk nutrient concentrations at the biofilm-stool interface were set as $N_{b,i}(0)$, while bulk concentrations at the intestine-biofilm interface $N_{b,i}(L)$ were assumed to be zero for simplicity. The byproduct transport equations and boundary conditions had a similar form,

$$\frac{\partial P_i}{\partial t} = \sum_{j=1}^{n} v_{i,j} X_j + D_{P,i}\frac{\partial^2 P_i}{\partial z^2}, \tag{5}$$

$$-D_{P,i}\frac{\partial P_i(0,t)}{\partial z} = k_{P,i}[P_{b,i}(0) - P_i(0,t)], \quad -D_{P,i}\frac{\partial P_i(L,t)}{\partial z} = k_{P,i}[P_{b,i}(L) - P_i(L,t)], \tag{6}$$

where $P_i(z,t)$ is the local concentration (mmol/L) of byproduct i (acetate, butyrate, CO_2, ethanol, formate, lactate, propionate, succinate), $D_{P,i}$ is a byproduct diffusion coefficient, $k_{P,i}$ is a byproduct mass transfer coefficient, $P_{b,i}(0)$ and $P_{b,i}(L)$ are bulk concentrations of byproduct i, which were assumed to be zero at both boundaries for simplicity. The exchange flux $v_{i,j}(z,t)$ (mmol/gDW/h) of product i from species j was positive if the byproduct was secreted and negative if the byproduct was consumed.

Each species was allowed to consume the four carbohydrates and the ten amino acids. Based on our previous study [2], we also included byproduct cross feeding between the species by allowing *F. prausnitzii* to consume acetate and succinate synthesized by *B. thetaiotaomicron* and *E. coli* and *B. thetaiotaomicron* to consume ethanol secreted by *E. coli* (see Figure 1B). Uptake kinetics for each nutrient and byproduct were assumed to follow Michaelis-Menten kinetics:

$$v_i = \frac{v_{max,i}S_i}{K_{m,i} + S_i} \tag{7}$$

where $S_i(z,t)$ is the local extracellular concentration (mmol/L) of substrate i (includes nutrients and byproducts) and $v_{max,i}$ and $K_{m,i}$ are Michaelis-Menten constants. The calculated local uptake rates $v_i(z,t)$ (mmol/gDW/h) were imposed as transport bounds in the linear program used to solve the metabolic reconstructions. Uptake kinetics did not account for catabolic repression by glucose because this regulatory effect [3] has not been well studies with respect to SCFAs and organic acids for gut anaerobes such as *B. thetaiotaomicron* and *F. prausnitzii* and glucose is the only carbon source for *E. coli*.

Model parameters

The simulated gut environment included nutrients that were assigned fixed uptake rate bounds and assumed not to limit growth. These components were sufficient to support *in silico* growth of all three species. FBA was used to find lower bounds that were non-limiting (see Additional Table 1).

The simulated gut environment also included nutrients (carbohydrates, amino acids, oxygen) that were assumed to limit growth. Local uptake rates of these components were calculated from local extracellular concentrations using Michaelis-Menten kinetics (see Equation 7). The bulk concentrations used to calculate these local concentrations (see Equation 4) were dependent on the assumed diet. The bulk concentrations used for each diet are listed in Additional Table 2. All three diets provided the same total carbon (6.5 mM) on a C6 basis.

Additional Table 1: Non-growth limiting media components with fixed uptake bounds. All bounds have units mmol/gDW/h.

Component	Bound	Component	Bound	Component	Bound
Biotin	-0.1	H+	-10	Ammonia	-10
Calcium	-0.1	H2O	-10	Protoheme	-0.1
Cob(I)alamin	-0.1	Hydrogen sulfide	-1	Phosphate	-10
Chloride	-0.1	K+	-0.1	(R)-Pantothenate	-0.1
Co2+	-0.1	Mg	-0.1	Pyridoxal	-0.1
Cu2+	-0.1	Mn2+	-0.1	Riboflavin	-0.1
Fe2+	-0.1	Molybdate	-0.1	Sulfate	-1
Fe3+	-0.1	Sodium	-0.1	Tungstate	-0.1
Folate	-0.1	Nicotinate	-0.1	Zinc	-0.1

Additional Table 2: Bulk carbohydrate and amino acid concentrations representing the three simulated diets. All concentrations have units mM.

**Nutrient**	**High Protein**	**Equal CHO-Protein**	**High CHO**
Arabinose	0.360	0.780	1.200
Fructose	0.300	0.650	1.000
Galactose	0.300	0.650	1.000
Glucose	0.600	1.300	2.000
Total CHO	1.560	3.380	5.200
Cysteine	1.000	0.650	0.300
Isoleucine	0.500	0.325	0.150
Leucine	0.500	0.325	0.150
Lysine	0.500	0.325	0.150
Methionine	0.600	0.390	0.180
Proline	0.600	0.390	0.180
Serine	1.000	0.650	0.300
Threonine	0.750	0.488	0.225
Tryptophan	0.273	0.177	0.082
Valine	0.600	0.390	0.180
Total AA	6.323	4.110	1.897

Model solution

DFBAlab [4] requires the specification of lexicographic optimization objectives to overcome the problem of alternative optima in the FBA problems. Because the biofilm model included three species and $N = 20$ spatial node points, a total of 60 FBA problems were solved at each time point. We found that the use of lexicographic optimization was essential because alternative optima invariably occurred in multiple models at different times during a simulation. Following the procedure established in our previous study [2], we ordered the lexicographic optimization objectives in the following tiers (Additional Table 3): (1) growth; (2) secretion of experimentally observed byproducts: *B. thetaiotaomicron* (acetate, succinate, propionate, CO_2) [5], *F. prausnitzii* (lactate, butyrate, formate, CO_2) [6], *E. coli* (acetate, ethanol, formate, lactate, succinate, CO_2) [7]; (3) uptake of possible cross-fed byproducts: *B. thetaiotaomicron* (lactate, ethanol, formate), *F. prausnitzii* (acetate, succinate), *E. coli* (butyrate, propionate); (4) uptake of supplied carbohydrates (arabinose, fructose, galactose, glucose); (5) uptake of supplied oxygen; and (6) uptake of supplied amino acids (cysteine, isoleucine, leucine, lysine, methionine, proline serine, threonine, tryptophan, valine). All objectives were specified as maximization, which translated to maximization of secretion fluxes due to their positivity and minimization of uptake fluxes due

to their negativity. The *B. thetaiotaomicron* model had only 23 objectives due to the lack of butyrate metabolism, while the *F. prausnitzii* model had only 22 objectives due to the lack of ethanol and propionate metabolism.

Additional Table 3: Lexicographic optimization objectives.

**Objective**	***B. thetaiotaomicron***	***F. prausnitzii***	***E. coli***
1	Biomass	Biomass	Biomass
2	Acetate	Lactate	Acetate
3	Succinate	Butyrate	Ethanol
4	Propionate	Formate	Formate
5	CO_2	CO_2	Lactate
6	Lactate	Acetate	Succinate
7	Ethanol	Succinate	CO_2
8	Formate	Arabinose	Butyrate
9	Arabinose	Fructose	Propionate
10	Fructose	Galactose	Arabinose
11	Galactose	Glucose	Fructose
12	Glucose	Oxygen	Galactose
13	Oxygen	Cysteine	Glucose
14	Cysteine	Isoleucine	Oxygen
15	Isoleucine	Leucine	Cysteine
16	Leucine	Lysine	Isoleucine
17	Lysine	Methionine	Leucine
18	Methionine	Proline	Lysine
19	Proline	Serine	Methionine
20	Serine	Threonine	Proline
21	Threonine	Tryptophan	Serine
22	Tryptophan	Valine	Threonine
23	Valine	Not specified	Tryptophan
24	Not specified	Not specified	Valine

Incorporation of Host-Microbiota Feedback

Our biofilm model predicted how oxygen affected species abundances and metabolite levels, but the model did not include how these variables affected oxygen levels resulting from host inflammation. A simple linear relationship between the *F. prausnitzii* biomass concentration averaged across the biofilm ($\bar{X}_{FP}$) and the bulk oxygen concentration ($O_{2,b}$) was developed to account for bidirectional host-microbiota interactions. This relationship was motivated by numerous studies demonstrating the anti-inflammation properties of butyrate and other compounds secreted from *F. prausnitzii* [8, 9, 10]. The linear equation was formulated using a Lagrange polynomial to interpolate the following two points for $[\bar{X}_{FP}, O_{2,b}]$: $[\bar{X}_{FP,anaerobic}, O_{2,perturb}]$ and $[0, O_{2,max}]$. The first point represented a sustained oxygen perturbation that occurred at time zero when the *F. prausnitzii* concentration was at its anaerobic value. The second point represented the maximum bulk oxygen concentration that would be attained for a zero *F. prausnitzii* concentration. We chose $O_{2,max} = 5x10^{-3}$ mM since this bulk concentration completely eliminated *F. prausnitzii* for the high CHO diet (see Figure 3D). The time-dependent interpolation polynomial was:

$$O_{2,b}(t) = \frac{\bar{X}_{FP}(t) - \bar{X}_{FP,anaerobic}}{0 - \bar{X}_{FP,anaerobic}} O_{2,max} + \frac{\bar{X}_{FP}(t) - 0}{\bar{X}_{FP,anaerobic} - 0} O_{2,perturb} \quad (8)$$

This equation is plotted for the CHO diet and three different values of $O_{2,perturb}$ in Figure 6A.

Incorporation of Antibiotics

Our biofilm model did not include antibiotics and their effects on the gut microbiota. To study the combined effects of antibiotics and oxygen, we modified the species biomass equations to include antibiotic mediated cell death. The modification was based on several simplifying assumptions including: (1) antibiotic pharmacokinetics was neglected; (2) the antibiotic concentration was constant throughout the biofilm due to the rapid release rate of antibiotic from cells compared to the antibiotic diffusional dynamics; (3) the antibiotic targeted growing cells such that the death rate was proportional to the species growth rate μ_i; and (4) the death rate was proportional to the specified antibiotic concentration A and the local biomass concentration X_i. The modified version of the biomass equation (1) was,

$$\frac{\partial X_i}{\partial t} = \mu_i X_i - k_d \mu_i X_i A + D_{X,i} \frac{\partial^2 X_i}{\partial z^2} \tag{9}$$

The host-microbiota feedback relationship (8) was included without oxygen perturbation ($O_{2,perturb} = 0$) such that increased oxygen levels only could result from decreases in *F. prausnitzii* concentration. The death rate constant was chosen as $k_d = 250$ mM^{-1} such that the antibiotic would exert its effect at reasonable antibiotic concentrations of $A \sim 1x10^{-3}$ mM.

References

[1] Horn, H., Lackner, S.: Modeling of biofilm systems: a review. In: Productive Biofilms, pp. 53–76 (2014)

[2] Henson, M.A., Phalak, P.: Byproduct cross feeding and community stability in an in silico biofilm model of the gut microbiome. Processes **5**(1), 13 (2017)

[3] Brückner, R., Titgemeyer, F.: Carbon catabolite repression in bacteria: choice of the carbon source and autoregulatory limitation of sugar utilization. FEMS microbiology letters **209**(2), 141–148 (2002)

[4] Gomez, J.A., Hoffner, K., Barton, P.I.: DFBAlab: a fast and reliable MATLAB code for dynamic flux balance analysis. BMC bioinformatics **15** (2014)

[5] Heinken, A., Sahoo, S., Fleming, R.M., Thiele, I.: Systems-level characterization of a host-microbe metabolic symbiosis in the mammalian gut. Gut microbes **4**(1), 28–40 (2013)

[6] Heinken, A., Khan, M.T., Paglia, G., Rodionov, D.A., Harmsen, H.J., Thiele, I.: Functional metabolic map of *Faecalibacterium prausnitzii*, a beneficial human gut microbe. Journal of bacteriology **196**(18), 3289–3302 (2014)

[7] Baumler, D.J., Peplinski, R.G., Reed, J.L., Glasner, J.D., Perna, N.T.: The evolution of metabolic networks of *E. coli.* BMC systems biology **5**(1), 182 (2011)

[8] Khan, M.T., Duncan, S.H., Stams, A.J., Van Dijl, J.M., Flint, H.J., Harmsen, H.J.: The gut anaerobe *Faecalibacterium prausnitzii* uses an extracellular electron shuttle to grow at oxic–anoxic interphases. The ISME journal **6**(8), 1578 (2012)

[9] Rossi, O., Van Berkel, L.A., Chain, F., Khan, M.T., Taverne, N., Sokol, H., Duncan, S.H., Flint, H.J., Harmsen, H.J., Langella, P., *et al.*: *Faecalibacterium prausnitzii* a2-165 has a high capacity to induce il-10 in human and murine dendritic cells and modulates t cell responses. Scientific reports **6**, 18507 (2016)

[10] Sokol, H., Pigneur, B., Watterlot, L., Lakhdari, O., Bermúdez-Humarán, L.G., Gratadoux, J.-J., Blugeon, S., Bridonneau, C., Furet, J.-P., Corthier, G., *et al.*: *Faecalibacterium prausnitzii* is an anti-inflammatory commensal bacterium identified by gut microbiota analysis of Crohn's disease patients. Proceedings of the National Academy of Sciences **105**(43), 16731–16736 (2008)
