# Supplementary material for: Microbiota dysbiosis in inflammatory bowel diseases: in silico investigation of the oxygen hypothesis
Source: BMC Syst Biol. 2017 Dec 28;11:145. doi: 10.1186/s12918-017-0522-1 (PMC5745886; doi:10.1186/s12918-017-0522-1)
Supplement: Supplementary file 2 — Pdf file for other simulation results. (PDF 181 kb) [file 12918_2017_522_MOESM2_ESM.pdf]

Additional file 2 — Other Simulation Results

We performed anaerobic simulations with the CHO diet to determine the minimum number of spatial discretization node points N needed for a satisfactorily accurate numerical solution. The solution was considered accurate if the average error of every state variable averaged across the biofilm at steady state was less than 0.5%. Since the true solution was not known, the error was computed with respect to the solution obtained for the largest $N = 30$. The solution appeared to be nearly converged with $N = 20$ where the largest error for any variable was less than 0.3% (Additional Figure 1A). Given that $N = 20$ required only 50% of the CPU time of $N = 30$ (Additional Figure 1B), we concluded that $N = 20$ provided a suitable compromise between numerical accuracy and efficiency.

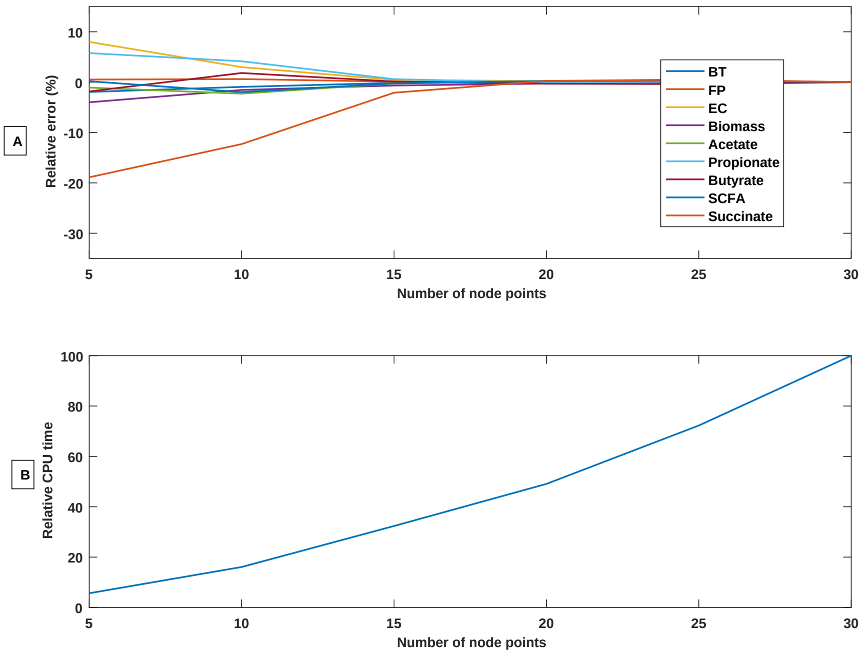

Additional Figure 1: Effect of the number of spatial discretization node points N on numerical solution accuracy. Anaerobic simulations were performed for the CHO diet and steady-state solution accuracy was calculated relative to the solution for $N = 30$. (A) Steady-state errors for species, total biomass, SCFA, total SCFA and succinate concentrations. (B) CPU time for a 10,000 hour dynamic simulation relative to the time required for $N = 30$.

We performed simulations to determine the impact of removing the extracellular electron shuttle *F. prausnitzii* uses to transfer electrons to oxygen and enable aerobic growth. The shuttle was eliminated by setting the upper bound

of the extracellular flavin reductase reaction (FLVXre) to zero in the *F. prausnitzii* genome-scale metabolic reconstruction [1]. Simulations were performed with the CHO diet over a range of bulk oxygen concentrations and compared to simulations with the shuttle active. As expected, removal of the shuttle effectively eliminated *F. prausnitzii* oxygen uptake (Additional Figure 2A). Without the ability to utilize oxygen as an electron acceptor and generate NAD+ through the FLVXre reaction, *F. prausnitzii* was less capable of competing with the other two species as the bulk oxygen concentration was increased (Additional Figure 2B–D). However the difference was not dramatic, suggesting that unmodeled *F. prausnitzii* oxygen toxicity might be more important than the inability to metabolize oxygen.

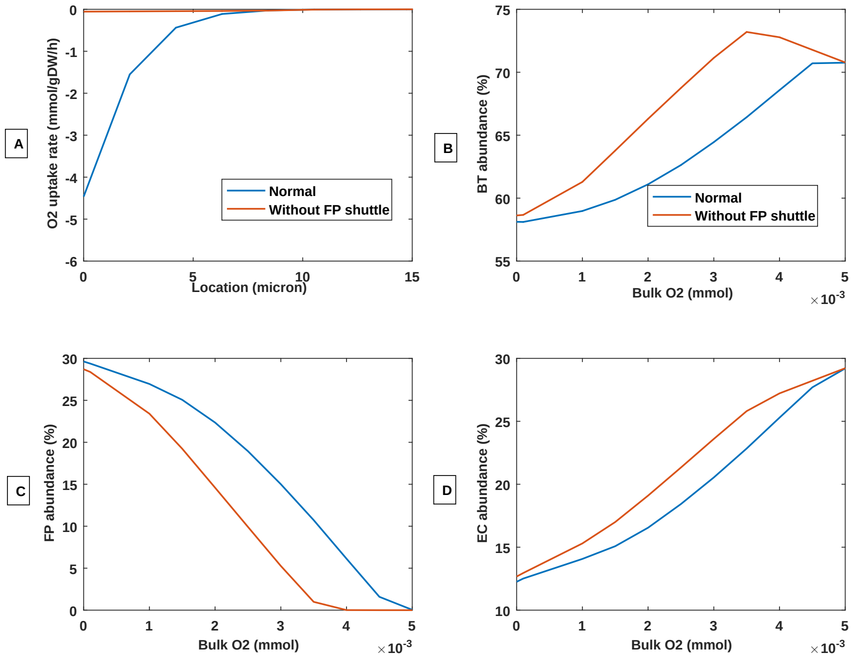

Additional Figure 2: The effect of removing the *F. prausnitzii* extracellular electron shuttle. Simulations were performed with the CHO diet and the upper bound of the extracellular flavin reductase reaction (FLVXre) set to zero. (A) Steady-state *F. prausnitzii* oxygen uptake with and without the shuttle active. ((B)–(D) Steady-state species abundances averaged across the biofilm with and without the shuttle active.

We performed simulations to explore the impact of sustained antibiotic treatment. The biomass equation for each species was modified to include an antibiotic mediated death term (Equation 9, additional file 1) assuming the antibiotic level was constant across the biofilm at a specified concentration A. The host-

microbiota feedback equation (Equation 8, additional file 1) with $O_{2,perturb} = 0$ was included so increased oxygen levels only could result from decreases in the *F. prausnitzii* concentration. Very little effect on species abundances (Additional Figure 3B–D) was predicted with $A = 0.5x10^{-3}$ mM as the positive feedback between *F. prausnitzii* and oxygen (Additional Figure 3A) was not strongly activated. A more pronounced effect was observed for $A = 0.85x10^{-3}$ mM, with the *F. prausnitzii* abundance decreasing 28% and the average oxygen level rising above 40 nM. When the antibiotic concentration was increased slightly to $A = 1x10^{-3}$ mM, the model predicted severe dysbiosis with *F. prausnitzii* essentially eliminated and the *E. coli* abundance increasing 107% from the baseline value. These predictions support the hypothesis that antibiotics can play a role in microbiota dysbiosis that characterizes IBD pathogenesis.

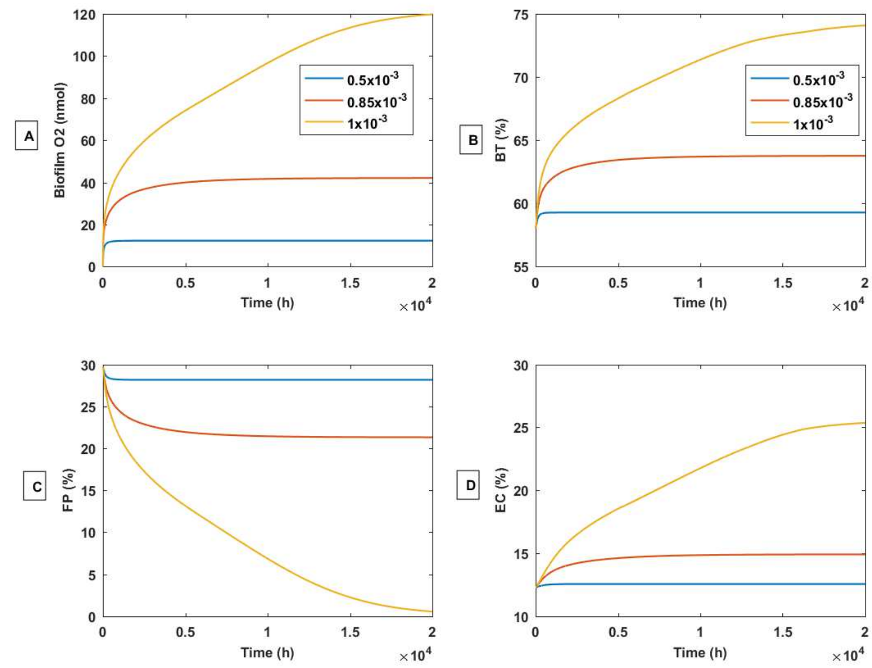

Additional Figure 3: The effect of sustained antibiotic treatment. Simulations were performed with the high CHO diet and the species biomass equations were modified to include an antibiotic mediated death term driven by a fixed antibiotic concentration A. (A) Oxygen concentration averaged across the biofilm for three A values. (B)–(D) Species abundances averaged across the biofilm for three A values.

References

[1] Heinken, A., Khan, M.T., Paglia, G., Rodionov, D.A., Harmsen, H.J.,

Thiele, I.: Functional metabolic map of *Faecalibacterium prausnitzii*, a beneficial human gut microbe. Journal of bacteriology **196**(18), 3289–3302 (2014)
